# Flexible Ultrahigh-Temperature Polymer-Based Dielectrics with High Permittivity for Film Capacitor Applications

**DOI:** 10.3390/polym9110596

**Published:** 2017-11-10

**Authors:** Zejun Pu, Xiaoyi Zheng, Yuhan Tian, Linqing Hu, Jiachun Zhong

**Affiliations:** College of Materials Science and Engineering, Sichuan University of Science & Engineering, Zigong 643000, China; a920036252@163.com (X.Z.); m18381306371@163.com (Y.T.); 18381307059@163.com (L.H.)

**Keywords:** polyarylene ether nitrile, surface functionalization, cross-linking, dielectric properties, energy storage capacity

## Abstract

In this report, flexible cross-linked polyarylene ether nitrile/functionalized barium titanate(CPEN/F-BaTiO_3_) dielectrics films with high permittivitywere prepared and characterized. The effects of both the F-BaTiO_3_ and matrix curing on the mechanical, thermal and dielectric properties of the CPEN/F-BaTiO_3_ dielectric films were investigated in detail. Compared to pristine BaTiO_3_, the surface modified BaTiO_3_ particles effectively improved their dispersibility and interfacial adhesion in the polymer matrix. Moreover, the introduction of F-BaTiO_3_ particles enhanced dielectric properties of the composites, with a relatively high permittivity of 15.2 and a quite low loss tangent of 0.022 (1 kHz) when particle contents of 40 wt % were utilized. In addition, the cyano (–CN) groups of functional layer also can serve as potential sites for cross-linking with polyarylene ether nitrile terminated phthalonitrile (PEN-Ph) matrix and make it transform from thermoplastic to thermosetting. Comparing with the pure PEN-ph film, the latter results indicated that the formation of cross-linked network in the polymer-based system resulted in increased tensile strength by ~67%, improved glass transition temperature (*T*_g_) by ~190 °C. More importantly, the CPEN/F-BaTiO_3_ composite films filled with 30 wt % F-BaTiO_3_ particles showed greater energy density by nearly 190% when compared to pure CPEN film. These findings enable broader applications of PEN-based composites in high-performance electronics and energy storage devices materials used at high temperature.

## 1. Introduction

As one of the most promising high-performance polymers, polyarylene ether nitrile (PEN) has attracted extensive attention in recent years owing to its outstanding properties, including excellent mechanical properties, high thermal stability, radiation resistance and chemical inertia [1,2]. These characteristics make it a good candidate to be widely used in various fields, including electronics and electrical appliances, automobiles, outer space, etc. Specifically, the possible applications of PEN as dielectric materials have been widely explored [3,4]. Although the abundant existence of strongly polar nitrile groups (–CN) pendant on the side-chain of PEN can promote the dielectric properties of PEN effectively, the low inherent permittivity (<5) and glass transition temperature (under 250 °C) of PEN still impede their use for high-k applications in electrical equipment encountered with extremely high temperature [5]. As we all know, it is effective to enhance the dielectric properties by incorporating PEN with a variety of inorganic conductive fillers, including polyaniline [6], carbon nanotubes [7], graphene [8], etc. Although this kind of PEN composites may possess higher permittivity, their intrinsic percolation characteristics resulted in higher dielectric loss and significantly lower breakdown strength [9]. Therefore, more attention to achieve synchronous improvement of permittivity and breakdown strength has been focused on fabrication of PEN composite dielectrics.

In recent years, other strategies, such as blending of polymer with high-k ceramic powder, chemical modification and cross-linking, have been widely adopted to obtain the high-performance PEN composite dielectrics. Tang et al. [10] fabricated core-shell structured BaTiO_3_@polymer (F-BaTiO_3_) nanoparticles through chemical grafting carboxyl-functionalized polyarylene ether nitrile (PEN–COOH) as the coating layer, and the obtained F-BaTiO_3_/PEN nanocomposites containing 40 wt % F-BaTiO_3_ loading content possess a dielectric constant of 13 and loss tangent of 0.023. Xie et al. [11] reported the fabrication of stretchable PVDF-based composite films by using surface modification of BaTiO_3_ as fillers and cross-linked PVDF as the polymer matrix, and the mechanical properties and energy storage capacities of the obtained c-PVDF/PDA@BaTiO_3_ were obviously improved when compared to pristineBaTiO_3_ filled PVDF composites. Yang et al. [12] grafted 4-nitrophthalonitrile onto the ends of PEN polymer chains, and then prepared polyarylene ether nitrile terminated phthalonitrile (PEN-Ph) film combined with post curing at high temperature. Through post curing treatment, the cross-linked PEN film possesses excellent mechanical properties and ultrahigh glass transition temperature up to 380 °C. These researches revealed that introducing cross-links and ferroelectric ceramic particles with high-k into PEN matrix would dramatically improve their comprehensive performance.

As a typical dielectric ceramic, barium titanate (BaTiO_3_) is used extensively to fabricate polymer/ceramic composite dielectrics due to its superior dielectric constants (ε > 10^3^) and low loss tangent [13,14,15,16,17]. However, poor dispersion and interfacial adhesion of BaTiO_3_ particles cause them to become easily agglomerated. In addition, as the fillers content is increased to a high level, e.g., 50 vol %, the uneven electrical field distribution and deteriorated flexibility would have negative influences on the dielectric properties and breakdown strength of the polymer/ceramic composites. In the previous studies, carboxyl-functionalized polyarylene ether nitrile (PEN–COOH), a thermoplastic polymer with a large number of carboxyl groups (–COOH), was employed to coat shielding layer on BaTiO_3_ particles surface [18]. Consequently, the modified BaTiO_3_ particles effectively improved their compatibility and dispersibility in the polymer matrix. In addition, to obtain high performance PEN-sPh cross-linked films, PEN-ph is synthesized according to the previously reported method [19]. Through simply post-curing at high temperature, the thermal stability of PEN-ph composites can be improved by a large margin. Therefore, by combining their respective advantages of surface modified BaTiO_3_ particles and cross-linkable PEN-ph, a kind of polymer/ceramic composite dielectrics could be expected. 

In this work, we report the fabrication of flexible cross-linked polyarylene ether nitrile/functionalized BaTiO_3_ (CPEN/F-BaTiO_3_) composite films with high permittivity. BaTiO_3_ particles were first chemically modified with PEN–COOH to form the shielding layers on their surface. To obtain composites with enhanced dispersibility and interfacial adhesion between two phases, the mixed solutionofF-BaTiO_3_ and PEN-ph were precipitated in the as-prepared solution (*V*_DIW_/*V*_NMP_ = 1:1) by using a high-speed shear dispersing machine to obtain homogeneously dispersed powder. Then, the PEN-ph/F-BaTiO_3_ dielectrics films were prepared by using F-BaTiO_3_ particles as fillers and PEN-ph as polymer matrix under melt processing. After further chemical cross-linking at high temperature, the flexible CPEN/F-BaTiO_3_ composite films were successfully fabricated. The combined influence of F-BaTiO_3_ and chemical cross-linking on the properties of CPEN/F-BaTiO_3_ composite films were investigated in detail.

## 2. Experimental Section

### 2.1. Materials

**Figure 1:**
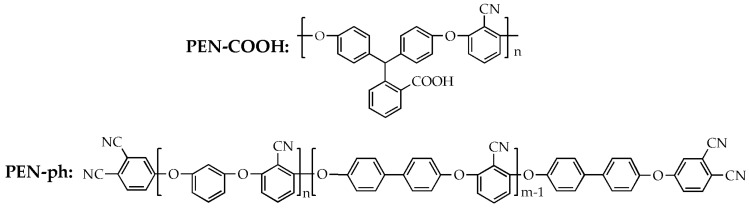
The structures of carboxyl-functionalized polyarylene ether nitrile (PEN–COOH) and polyarylene ether nitrile terminated with phthalonitrile (PEN-ph).

Barium titanate (BaTiO_3_) (the diameter: 80 ± 10 nm; purity: 99.9%) was purchased from Shanghai Aladdin biochemical technology Co. Ltd., Shanghai, China. In addition, 2,6-dichlorobenzonitrile (DCBN) was purchased from Yangzhou tianchen fine chemical Co. Ltd., Yangzhou, China. Hydroquinone (HQ), biphenyl (BP) and potassium carbonate (K_2_CO_3_) were purchased from Kelong reagent Co. Ltd., Chengdu, China. All the materials were used without further purification. The structures of carboxyl-functionalized polyarylene ether nitrile (PEN–COOH) and polyarylene ether nitrile terminated with phthalonitrile (PEN-ph) are shown in Figure 1. PEN–COOH was synthesized in our laboratory according to the previously reported method [20]. PEN-ph was synthesized by the nucleophilic aromatic substitution polymerization of 2,6-dichlorobenzonitrile (DCBN), hydroquinone (HQ) and biphenyl (BP), followed by termination with 4-nitrophthalonitrile [21]. Then, PEN-ph product was purified several times by acetone and deionized water until impurities were washed out. The number average molecular weight of as-synthesized PEN–COOH and PEN-ph copolymer determined by GPC is 6 × 10^3^ and 3.4 × 10^4^, respectively. 

### 2.2. Preparation of F-BaTiO_3_ Particles

In this study, the BaTiO_3_ particles were surface modified using a carboxyl-functionalized polyarylene ether nitrile (PEN–COOH) as the surface-grafting agent. PEN–COOH, a thermoplastic polymer with a large number of carboxyl groups (–COOH), was employed due to its powerful ability to coat different thickness of polymer layers on nearly any oxide particles surface, such as SiO_2_, TiO_2_, Fe_3_O_4_, etc. This kind of strategy has already been verified its high-efficiency in improving dispersibility and compatibility of oxide particles with polymer matrix [18].

### 2.3. Preparation of the CPEN/F-BaTiO_3_ Composites Films

**Figure 2:**
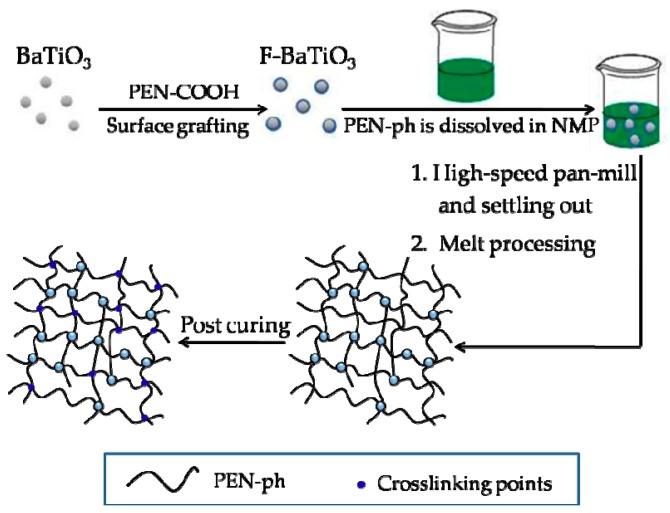
Schematic illustration for the surface modification of BaTiO_3_ particles and preparation of CPEN/F-BaTiO_3_ composite films.

The flexible cross-linked polyarylene ether nitrile/functionalized BaTiO_3_ (CPEN/F-BaTiO_3_) composite films with high permittivitywere fabricated and illustrated in Figure 2. Firstly, the mixed solution of F-BaTiO_3_ and PEN-ph were precipitated in the as-prepared solution (*V*_DIW_/*V*_NMP_ = 1:1) by a high-speed shear dispersing machine to obtain homogeneously dispersed powder. Then, the CPEN/F-BaTiO_3_ composite films with the thickness of ~60 μm were fabricated by melt processing of as-prepared powder combined with post curing at high temperature (260 °C, 280 °C, 300 °C, 320 °C, 340 °C and 360 °C every for 3 h). The weight contents of F-BaTiO_3_ particles, 0, 10, 20, 30 and 40 wt %, was designed in resultant films and labeled as P-0, P-1, P-2, P-3 and P-4, respectively. Through post curing with high temperature, the phthalonitriles capped at the ends of PEN and the cyano (–CN) groups grafted on the surface of BaTiO_3_might form phthalocyanines or triazine ring as the cross-linking points in the system [22,23]. Therefore, the obtained cross-linking network would be of great advantage to enhance the thermal stability of the as-prepared composite films.

### 2.4. Characterization

The chemical composition of the samples was characterized by Fourier transform infrared spectra (FTIR, Shimadzu 8000S, Kyoto, Japan). The micro-morphologies of F-BaTiO_3_ particles and CPEN/F-BaTiO_3_ composite films were observed by transmission electron microscope (TEM, FEI Tecnai G2 F20, Hillsboro, OR, USA) and scanning electron microscope (SEM, FEI INSPECT F50, Hillsboro, OR, USA). The crystal structures of pristine BaTiO_3_ and F-BaTiO_3_ were characterized by X-ray diffraction (XRD, Rigaku, Tokyo, Japan, RINI2400 with Cu Kα radiation). Mechanical properties of the composite films were performed using a SANS CMT6104 series desktop electromechanical universal testing machine (Shenzhen Sans Materials Testing Machine Co., Shenzhen, China), and the stretching speed was 5mm/min. All samples (with the sample size as 10 × 100 mm) were tested at room temperature and reported as an average value for every five samples. Dynamic mechanical analysis (DMA) was carried out on TA instrument (TA-Q800, TA Instruments Ltd., New Castle, DE, USA). Dielectric properties for the composite films were monitored according to the ASTM D150 on a TH 2819A precision LCR meter (Tonghui Electronic Co., Ltd., Dongguan, China). Before testing, conductive silver paste was brushed to a specific area on both sides of the samples to form a plate capacitor. The breakdown strength for PEN-based composite films was tested by a dc dielectric withstand voltage tester (catic era instrument equipment Co., Ltd., Beijing, China) in an oil bath. The applied voltage began at 40 V and increased at approximately 0.1 KV/s until the breakdown failure of a sudden current increase. The reported values were calculated as average values by five samples for each film.

## 3. Results and Discussion

### 3.1. Characterizations of F-BaTiO_3_ Particles

**Figure 3:**
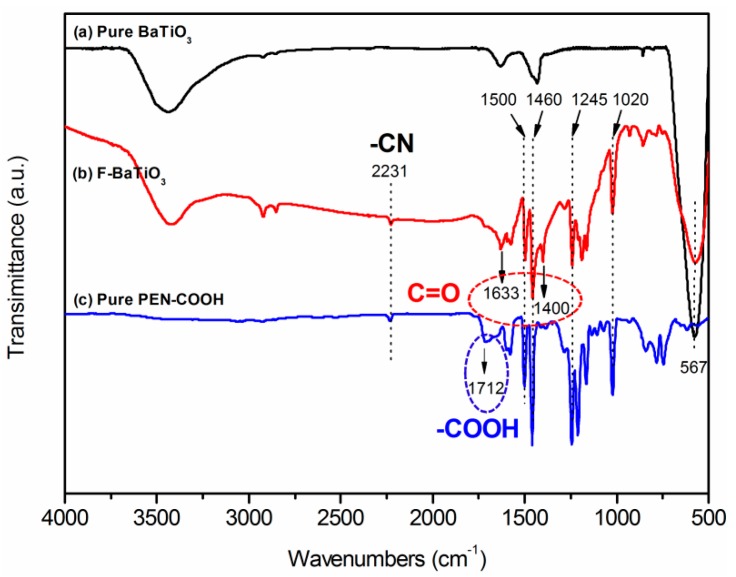
FTIR spectra of: (**a**) pure BaTiO_3_; (**b**) F-BaTiO_3_; and (**c**) pure PEN–COOH.

The chemical composition and morphology of F-BaTiO_3_ particles were characterized by FTIR, TEM and XRD, respectively. Firstly, the surface modifying of BaTiO_3_ particles was verified by FTIR. As shown in Figure 3, a strong absorption peak appeared at 567 cm^−1^, which corresponds to the Ti-O vibration. Compared to pristine BaTiO_3_, two new absorption bands appeared at 1460 cm^−1^ and 1500 cm^−1^ in F-BaTiO_3_ belongs to the skeleton vibration of benzene rings in PEN–COOH. Moreover, the characteristic absorption peaks at 1022 and 1245 cm^−1^ belong to the symmetric and asymmetric stretching vibrations of aryl ether, respectively. In addition, the new absorption band at 2231 cm^−1^ in F-BaTiO_3_ is mainly attributed to the symmetrical stretching vibration of nitrile groups (–CN). These results indicated the existence of PEN–COOH in F-BaTiO_3_ particles. It is worth noting that the peak at 1712 cm^−1^ belonging to carboxyl groups (–COOH) in PEN–COOH is not observed in the F-BaTiO_3_ particles. However, the new absorption bands at 1400 and 1633 cm^−1^ in F-BaTiO_3_are mainly attributed to the symmetric and asymmetric stretching vibrations of carbonyl groups (C=O), respectively. The latter suggested the successful chemical grafting of PEN–COOH onto the surfaces of BaTiO_3_ particles [24].

**Figure 4:**
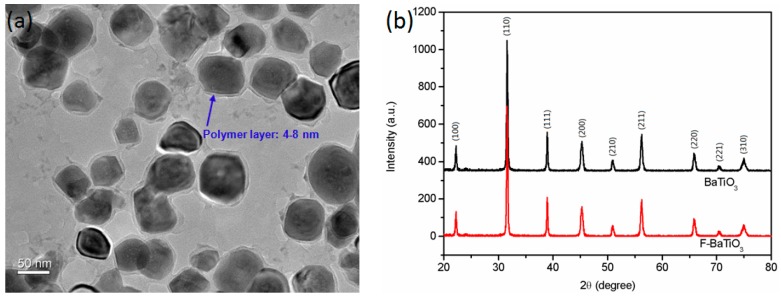
(**a**) TEM images of F-BaTiO_3_ particles; and (**b**) XRD spectra of pristine BaTiO_3_ and F-BaTiO_3_ particles.

TEM scanning is the visualized observation for the coating structure of PEN–COOH around BaTiO_3_ particles. Figure 4a depicts a distinct contrast between PEN–COOH shell and BaTiO_3_ core. It can be clearly seen that the outermost light-colored layer belongs to the PEN–COOH layer, which grafted on the surface of BaTiO_3_ particles with a uniform thickness of 4–8 nm. The XRD spectra of pristine BaTiO_3_ and F-BaTiO_3_ particles are shown in Figure 4b. These diffraction peaks at 2θ = 22.2°, 31.6°, 38.9°, 45.3°, 50.9, 56.3°, 65.9°, 70.4° and 74.8° are clearly observed, which can be indexed to the pristine BaTiO_3_ crystal plane of (100), (110), (111), (200), (210), (211), (220), (221) and (310), respectively. Obviously, all of the characteristic diffraction peaks of F-BaTiO_3_matched well with the curve of pristine BaTiO_3_, which is well consistent with the standard cards (JCPDS 31-0174) [25]. Moreover, for these surface grafted F-BaTiO_3_ particles, superfluous peaks cannot be detected, indicating the well-organized cubic crystal form [26].

### 3.2. Chemical Structure and Morphology of CPEN Composite Films

**Figure 5:**
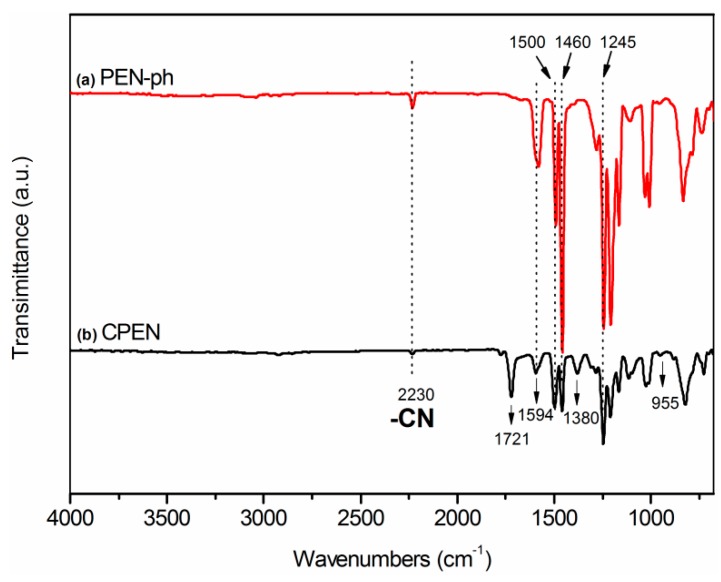
FTIR spectra of: (**a**) PEN-ph; and (**b**) CPEN films.

The chemical structure of the PEN-ph and CPEN films was characterized by FTIR. As shown in Figure 5, it is observed that three absorption bands appear at 1594 cm^−1^, 1500 cm^−1^ and 1460 cm^−1^, which are attributed to skeleton vibration of benzene rings. The absorption band at 1245 cm^−1^ is clearly observed, which belonged to the absorption peak of aryl ether. The characteristic band at 2230 cm^−1^ is coming from the absorption peak of cyano groups (–CN). A comparison with the PEN-ph film appeared some new absorption peaks in CPEN film, meaning that the formation of cross-linked network structure of the CPEN resin. Apparently, the absorption peak at 2231 cm^−1^ gradually weakened with curing treatment. However, the new absorption bands at 1721 and 1380 cm^−1^ are clearly observed in CPEN film, which are assigned to the characteristic peak of indole ring and triazine ring [27], respectively. In addition, the peak at 955 cm^−1^ belongs to the in-plane bending vibration of C–N. These results further indicated that the cross-linking of CPEN was successfully realized at high temperature, and formed the cross-linked network structure in the CPEN matrix. Furthermore, the cross-linking degree of the fragmented pure PEN-ph and CPEN film were measured through Soxhlet extraction, using NMP as solvent. Samples were refluxed in NMP for 48 h. It can be clearly observed that the fragmented PEN-ph film is completely dissolved in NMP. However, the fragmented CPEN film almost maintains a constant weight. The results indicated that the cross-linking degree of CPEN film is almost 100%, thus improving the thermal stability of the CPEN film.

**Figure 6:**
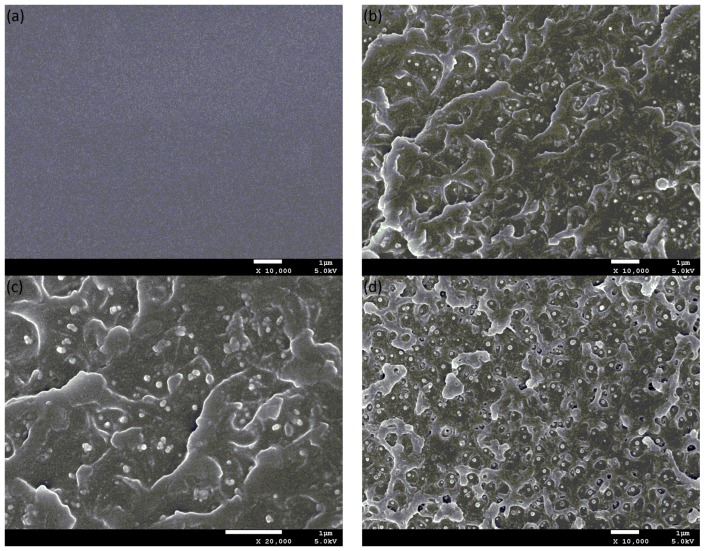
Cross-sectional SEM images of CPEN composite films with different F-BaTiO_3_ loading: (**a**) 0 wt %; and (**b**) 20 wt %; (**c**) enlarged details of (**b**); and (**d**) CPEN composite films with 20 wt % pristine BaTiO_3_ loading.

The cross-section morphologies of CPEN composite films were investigated through SEM, as presented in Figure 6. After the post curing, the pure CPEN film becomes rigid compared with pure PEN-ph film. In addition, the pure CPEN film showed homogeneous and smooth fracture morphology (Figure 6a), suggesting typical brittle fractures of a thermosetting material. Besides, the dispersibility and interfacial compatibility of fillers in CPEN matrix can also be clearly observed from the cross-sectional SEM images. As shown in Figure 6b, the 20 wt % F-BaTiO_3_ filled composite film displays relatively rough surface morphologies with wave-like structures. Importantly, there is almost no BaTiO_3_ particle agglomeration and the F-BaTiO_3_ particles are perfectly embedded in CPEN matrix. Besides, the enlarged detail of Figure 6b (see Figure 6c) shows that the interface between F-BaTiO_3_particles and CPEN matrix is indistinguishable, indicating improved interactions between the two phases through the PEN–COOH grafting. However, the comparison with F-BaTiO_3_ filled composite film revealed that the phase interface between pristine BaTiO_3_particles and CPEN matrix is fairly distinct, indicating a worse interfacial compatibility in this case (as seen from Figure 6d). These results can be attributed to the benefit of coating layer (PEN–COOH) on the surface of BaTiO_3_particles, improving the dispersal uniformity of BaTiO_3_ particles and promoting adhesion among BaTiO_3_ and PEN-ph matrix. Therefore, it can be predicted that the CPEN/F-BaTiO_3_ composite films will present excellent comprehensive performance, which is extremely important for expanding its practical application.

### 3.3. Mechanical Properties of CPEN Composite Films

**Figure 7:**
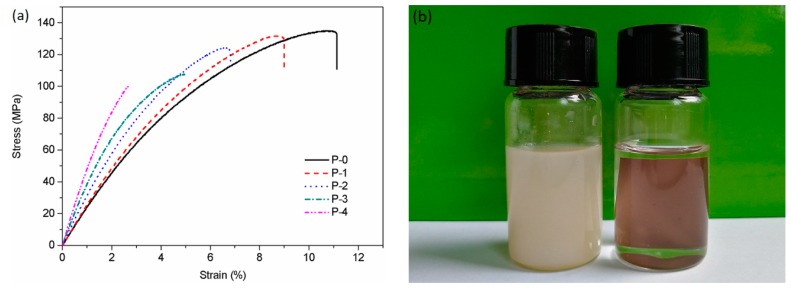
(**a**) The stress-strain curves of the CPEN/F-BaTiO_3_ composite films; and (**b**) digital figures of composite films with same filler loadings in NMP: uncross-linked (left); and cross-linked (right).

As a high-performance thermoplastic polymer, PEN-ph possesses outstanding properties including high thermal stability, excellent mechanical properties as well as chemical resistance [28,29,30]. Most importantly, the phthalonitrile is capped at the ends of PEN-ph can form cross-linked three-dimensional network structure at high temperature, thus further improving above-mentioned properties. The tensile strength and tensile modulus of pure PEN-ph film are 81 MPa and 2155 MPa, respectively [31]. After chemical cross-linking, the stress-strain curves of the CPEN/F-BaTiO_3_ composite films are shown in Figure 7a. It can be seen that the increase inF-BaTiO_3_ loading content raises Young’s modulus of all CPEN/F-BaTiO_3_ composite films. That is attributed to the contribution of ceramic particles on the modulus of all CPEN composite films. With increasing fillers content, the tensile strength decreases gradually but still higher than that of pure PEN-ph film. Comparing with the pure PEN-ph film, the tensile strength of P-0, P-1, P-2, P-3 and P-4 increased by 25%, 33%, 54%, 63% and 67%, respectively. The effective increase of the mechanical properties results from the increasing of cross-linking density to some extent. Besides, the elongation at break of the CPEN/F-BaTiO_3_ composite films continuously decreases with the increasing F-BaTiO_3_ loading content, declining to 2.7% from 11.1%. This similar phenomenon has widely been observed in many reported polymer/inorganic composite systems [32]. Figure 7b shows the chemical resistance of the composite films to a strong dissolving solvent, where PEN-ph/F-BaTiO_3_and CPEN/F-BaTiO_3_ composite films with 20 wt % fillers loading content were soaked in NMP and maintained for 4 h at 60 °C in an ultrasonic water bath. It is obviously observed that the PEN-ph/F-BaTiO_3_ film was completely dissolved and formed a uniform dispersion solution (Figure 7b, left). Particularly, the CPEN/F-BaTiO_3_ composite film actually maintained its original shape during the process (Figure 7b, right), which can be attributed to the chemically cross-linked PEN-ph matrix.

### 3.4. Dynamic Mechanical Properties of CPEN Composite Films

**Figure 8:**
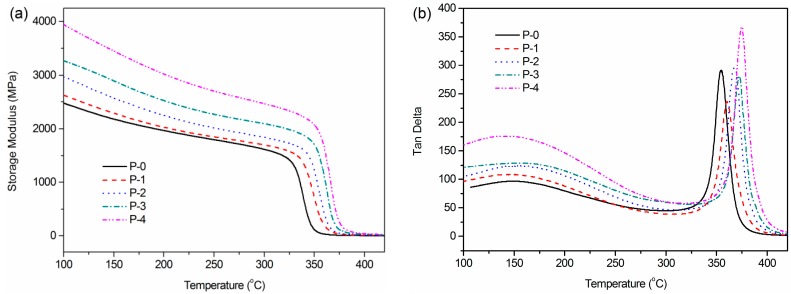
DMA measurement for CPEN/F-BaTiO_3_ composite films with different F-BaTiO_3_ loading: (**a**) the storage modulus; and (**b**) tanδ of composite films versus temperature.

The thermal properties of the CPEN composite films were investigated by means of dynamic mechanical analysis (DMA) and their DMA curves are shown in Figure 8. As can be seen, the storage modulus increases with the increasing of F-BaTiO_3_ loading content (see Figure 8a), which is attributed to the contribution of rigid particles (BaTiO_3_) on the modulus of all samples. Furthermore, the storage modulus gradually decreases as the temperature rises up to the onset of the glass relaxation, and then decreases abruptly during the glass relaxation, as expected. Importantly, it can be seen that all CPEN composite films present a damping peak for Tan delta in the range from 350 °C to 380 °C (as seen in Figure 8b), which corresponds to the glass transition temperature (*T*_g_) of the CPEN composite films. As can be seen, the *T*_g_ of the P-0 film is around 355 °C, which is higher than that of PEN-ph film (165 °C) [31]. In comparison with P-0 film, the *T*_g_s of P-1, P-2, P-3 and P-4 films are, respectively, increased by 6 °C, 13 °C, 18 °C and 20 °C. These results demonstrated that the *T*_g_s gradually increase as the degree of cross-linking reaction and F-BaTiO_3_ loading content increases. This may be attributed to three reasons as follows. Firstly, the film is self-cross-linked by the phthalonitrile groups capped at the ends of linear PEN-ph, which is the primary contributor of *T*_g_s increase. Secondly, it may be due to the improved dispersibility of filler and the strong physical entanglements of macromolecular chains between the functional intermediate PEN–COOH and PEN-ph matrix can reduce free volume and segmental mobility, thereby improving the *T*_g_. Thirdly, both PEN–COOH and PEN-ph have pendant nitrile groups (–CN), which can also form network structure between PEN–COOH and PEN-ph through chemical cross-linking. 

### 3.5. Dielectric Properties of CPEN Composite Films

**Figure 9:**
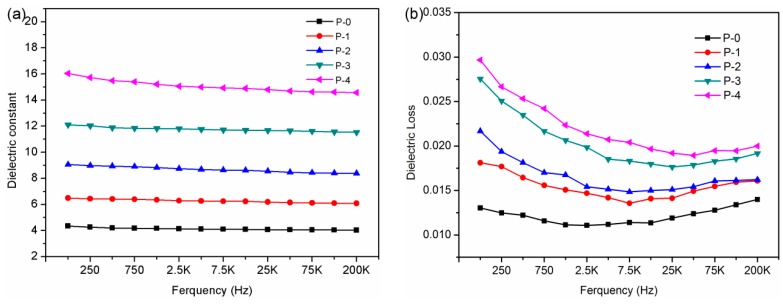
(**a**) Dielectric constant; and (**b**) loss tangent of CPEN/F-BaTiO_3_ composite films as a function of frequency.

The dielectric constant and loss tangent of CPEN/F-BaTiO_3_composite films were measured at room temperature as a function of frequency (Figure 9). The introduction of high-kF-BaTiO_3_ particles was the reason for the elevated dielectric constant, which has been well researched and finely predicted by using a series of parallel models [33,34]. For P-0 film, the dielectric constant (4.2) and loss tangent (0.011) are almost the same in the measured frequency range of 100 Hz–200 kHz. The dielectric constant of both composite films decreased slightly as a function of frequency, which is attributed to the effect of the polarization relaxation [35]. For P-1 composite film, the dielectric constant reached 6.4 at 1 kHz with an increment of 52% in comparison with that of P-0, which can be attributed to the introduction of high-kBaTiO_3_ particles. As shown in Figure 9a, it can be seen that the increase in F-BaTiO_3_ loading content raised nonlinearly the dielectric constant of all CPEN composite films. Specifically, the dielectric constant of P-4 composite film shows an about 4-fold increase at 1 kHz (up to 15.2 from 4.2 of P-0). Similar tendency can also be observed for the dielectric loss of the CPEN/F-BaTiO_3_ composite films (see from Figure 9b). Although the dielectric loss of CPEN/F-BaTiO_3_ composite films also increased with increasing content of F-BaTiO_3_, all of the CPEN/F-BaTiO_3_ composite films show relatively low dielectric loss, which is as low as 0.023 for P-4 at 1 kHz. Importantly, it is much lower than the F-BaTiO_3_/PEN composite at the same content of fillers reported in the reported literature [10].The result is because the F-BaTiO_3_particleshave good dispersibility and compatibility with the polymer matrix, which leads to a decrease of dielectric loss derived from interfacial polarization between inorganic and organic phases [1,33].

### 3.6. Electric Breakdown and Energy Storage Capacity of CPEN Composite Films

**Figure 10:**
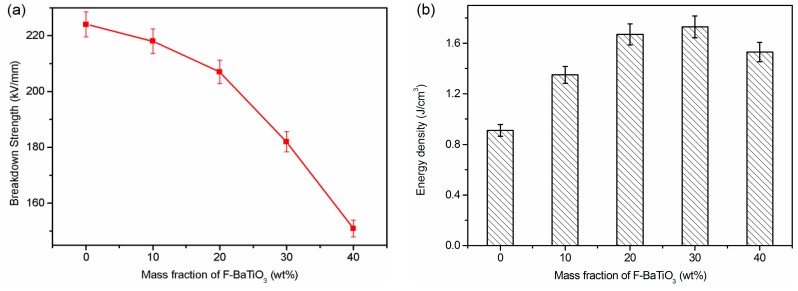
(**a**) The breakdown strength; and (**b**) energy density of the CPEN/F-BaTiO_3_ composite films at room temperature.

As indicated in the equation *U* = 1/2·ε_0_·ε_r_·(*E*_b_)^2^ (where ε_0_ is the vacuum dielectric constant with the value of 8.85 × 10^−12^ F/m, ε_r_ is the relative dielectric constant, and *E*_b_ is the dielectric breakdown strength), elevated breakdown strength of dielectric materials is rather important for achieving superior energy storage capacities. As can be seen from the above-mentioned equation, two variables (ε_r_ and *E*_b_) together determine the value of energy density. For the practical application in capacitor field, high permittivity and excellent breakdown strength of the materials are necessary. As can be seen in Figure 10a, the breakdown strength of the P-0 film is as high as 224 KV/mm, and a further increase in F-BaTiO_3_ loading content led to a gradual decrease in *E*_b_. As expected, the dielectric constant of both composites increases with the increasing F-BaTiO_3_ loading, and the increment of dielectric constant rises nonlinearly. As the F-BaTiO_3_ content increases to 30 wt %, the calculated results show that the P-3 composite film exhibits a maximum energy density of up to 1.73 J/cm^3^, which is 90% superior than the values obtained with P-0 film (0.91 J/cm^3^). This is the result of increase in dielectric constant and slight decrease in breakdown strength. With further increasing the F-BaTiO_3_ loading, although the dielectric constant still increases, the breakdown strength decreases dramatically, resulting in the decrease of energy storage density. The result is because high mass fraction of the F-BaTiO_3_ in the composite films may result in partial agglomeration of particles and increase in porosity, which could increase the uneven distribution of the electric field inside the composite films and weaken the breakdown strength [7,11,36].

## 4. Conclusions

Cross-linked polymer-based films as flexible dielectric materials that can be used at ultrahigh-temperature condition were prepared. BaTiO_3_ particles were firstly chemical modified with PEN–COOH to form the shielding layers on their surface. The F-BaTiO_3_ particles were characterized using FTIR, TEM and XRD. Then, the CPEN/F-BaTiO_3_ dielectric films with thickness of ~60 μm were fabricated by melt processing combined with post curing at high temperature. According to the results of DMA measurement, the CPEN/F-BaTiO_3_ composite films present ultra-high *T*_g_s (higher than 355 °C). Due to the chemical cross-linking, the mechanical properties of CPEN/F-BaTiO_3_ composite films were greatly enhanced. In addition, the composite films also show high breakdown strength, stable dielectric constant and low dielectric loss. As the mass fraction of F-BaTiO_3_ increases to 30 wt %, the calculated results indicate that the P-3 composite film exhibits a maximum energy density of up to 1.73 J/cm^3^, which is 90% better than the values obtained with P-0 film (0.91 J/cm^3^). In consideration of these advantages, the CPEN/F-BaTiO_3_ composite film would be a promising candidate as the dielectric materials used in high-performance electronic devices.
